# A Humanized Monoclonal Antibody Cocktail to Prevent Pulmonary Ricin Intoxication

**DOI:** 10.3390/toxins12040215

**Published:** 2020-03-29

**Authors:** Yinghui Rong, Michael Pauly, Adrian Guthals, Henry Pham, Dylan Ehrbar, Larry Zeitlin, Nicholas J. Mantis

**Affiliations:** 1 Division of Infectious Diseases, Wadsworth Center, New York State Department of Health, Albany, NY 12208, USA; yzr0002@tigermail.auburn.edu (Y.R.); dylan.ehrbar@health.ny.gov (D.E.); 2 Mapp Biopharmaceutical, Inc. 6160 Lusk Blvd, San Diego, CA 92121, USA; michael.pauly@mappbio.com (M.P.); adrian@guthals.com (A.G.); Henry.Pham@mappbio.com (H.P.); Larry.Zeitlin@mappbio.com (L.Z.)

**Keywords:** toxin, lung, antibody, biodefense, prophylactic

## Abstract

PB10 IgG_1_, a monoclonal antibody (MAb) directed against an immunodominant epitope on the enzymatic subunit (RTA) of ricin toxin (RT), has been shown to passively protect mice and non-human primates from an aerosolized lethal-dose RT challenge. However, it was recently demonstrated that the therapeutic efficacy of PB10 IgG1 is significantly improved when co-administered with a second MAb, SylH3, targeting RT’s binding subunit (RTB). Here we report that the PB10/SylH3 cocktail is also superior to PB10 alone when used as a pre-exposure prophylactic (PrEP) in a mouse model of intranasal RT challenge. The benefit of the PB10/SylH3 cocktail prompted us to engineer a humanized IgG1 version of SylH3 (huSylH3). The huPB10/huSylH3 cocktail proved highly efficacious in the mouse model, thereby opening the door to future testing in non-human primates.

## 1. Introduction

Ricin toxin (RT) is classified by the Centers for Disease Control and Prevention (CDC) as a biological threat agent because of its extreme toxicity following inhalation [1]. In experimental settings, mice and non-human primates (NHPs) challenged with 3–10 x LD_50_ of RT by aerosol succumb to intoxication within a 24–48 h period [2,3]. The underlying cause of death following pulmonary RT exposure is likely acute respiratory distress [3,4,5,6]. Alveolar macrophages (AM) are particularly susceptible to RT and are postulated to contribute to amplification of disease severity through the secretion of pro-inflammatory cytokines, which have the potential to sensitize airway epithelial cells to toxin-induced programmed cell death [7,8,9,10,11].

Intervention studies with toxin-neutralizing monoclonal antibodies (MAbs) in mice and NHPs have demonstrated the intoxication process can be reversed, but only if MAbs are administered within a short window after RT exposure [12]. In a recent study conducted in Rhesus macaques, the five animals that received a single intravenous fusion of a humanized MAb, huPB10, within 4 h after 3 x LD_50_ RT aerosol exposure survived toxin challenge, whereas only a single animal that received huPB10 at 12 h survived RT intoxication [12]. These results largely mimicked what had been previously reported in mouse studies with the murine version of PB10, demonstrating a degree of congruence between the two animal models.

Because of the short therapeutic window, the prospect of leveraging toxin-neutralizing MAbs as pre-exposure prophylactics (PrEP) is appealing. Towards this goal, we recently demonstrated that a single infusion (25 mg/kg) of an extended serum-half-life variant of huPB10 called PB10-LS was sufficient to protect Rhesus macaques, 28 days later, from an RT challenge by aerosol [13]. PB10, originally identified as a mouse MAb, is directed against an immunodominant epitope near RTA’s active site [14] and is proposed to neutralize RT by interfering with trafficking of the toxin from the plasma membrane to the trans Golgi network (TGN) [15]. Humanized PB10 and the PB10-LS were each expressed in a *Nicotiana benthamiana*-based manufacturing platform [16,17].

Despite the success of huPB10-LS in the NHP model, there is evidence from mice that the potency of PB10 is significantly improved when combined with a second MAb, called SylH3, targeting RT’s binding subunit, RTB [10]. In that study, mice treated with low-dose PB10 alone survived intranasal ricin challenge, but experienced weight loss, moderate pulmonary inflammation (e.g., elevated IL-1 and IL-6 levels, PMN influx), and apoptosis of lung macrophages. In contrast, mice treated with an equimolar ratio of PB10 and SylH3 were nearly impervious to the effects of pulmonary ricin toxin exposure.

We are particularly interested in exploring the intranasal (aerosol) route of MAb delivery for two reasons. First, locally delivered antibodies have the potential to intercept RT before it can access the respiratory mucosa, possibly dampening early pro-inflammatory responses that contribute to ARDS [5]. Poli and colleagues demonstrated more than 20 years ago that aerosolized delivery of polyclonal anti-ricin antibodies one hour before a subsequent aerosolized ricin challenge was able to protect mice from toxin-induced death [18]. In that study, antibody was delivered by small particle aerosol with a Collison nebulizer. Secondly, we are interested in the prospect of developing a self-administered (inhalable) immunoprophylactic capable of conferring immediate immunity to category B toxins like RT. Indeed, Respaud and colleagues have already described a drug delivery system for efficient alveolar delivery of a neutralizing MAb to treat pulmonary intoxication [19].

With this as the background, we sought to investigate the potential of PB10 and SylH3 to function as a PrEP and determine whether the combination of MAbs is superior to either of the individual MAbs at protecting mice from RT-induced morbidity and mortality.

## 2. Results

**Figure 1:**
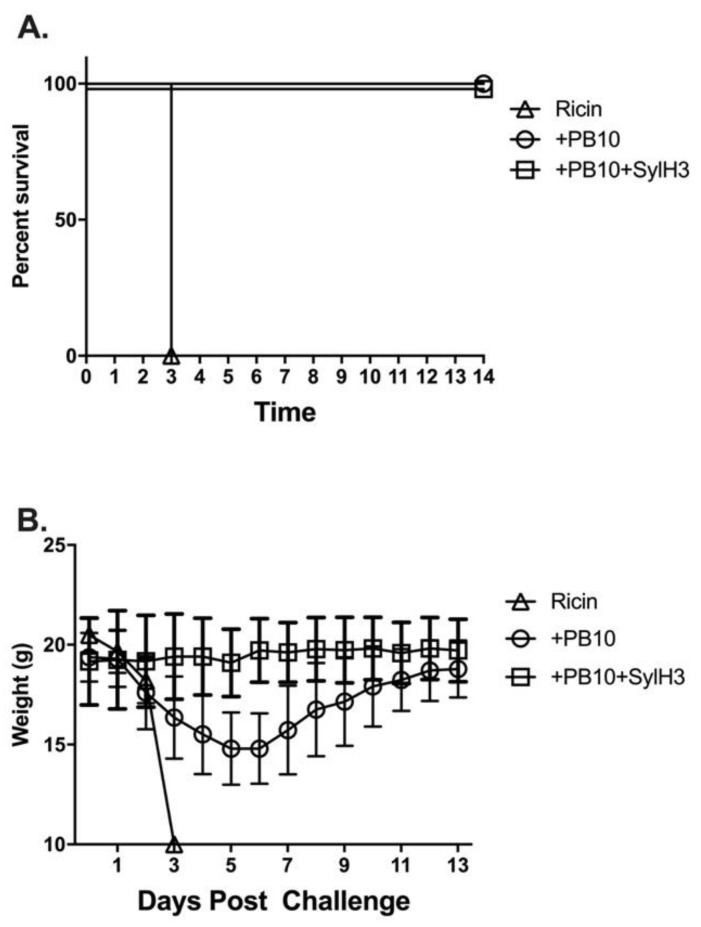
Benefit of PB10/SylH3 in protecting mice against intranasal ricin toxin (RT) exposure. Groups of mice received an intranasal instillation of PB10/SylH3 monoclonal antibody (Mab) cocktail (2 mg/kg), PB10 MAb (2 mg/kg) or vehicle (saline) 1 h before an RT challenge by the IN route. The dose of 2 mg/kg (40 µg per mouse) was chosen because previous studies had indicated that this was the minimum amount of antibody required to passively protect against intranasal ricin challenge. Following RT challenge, the mice were monitored for (**A**) survival and (**B**) weight loss for a period of 14 days, as described in the materials and methods section. The control animals that received saline prior to 10 x LD_50_ RT challenge experienced a rapid decline in body weight and expired (or were euthanized) within 72 h. Mice that received PB10 prior to RT challenge survived for the duration of the experiment (14 days) but experienced weight loss. In contrast, mice that were pre-treated with the PB10/SylH3 MAb cocktail survived RT challenge without a demonstrable change in weight, demonstrating that the PB10/SylH3 MAb cocktail is superior to PB10 when employed as a PrEP.

In this current report, we sought to examine whether the PB10/SylH3 cocktail affords a benefit over PB10 when administered to mice in advance of RT exposure. We first conducted a pilot study in which purified mouse PB10 (40 μg; 2 mg/kg) or PB10/SylH3 (20 μg PB10 + 20 μg SylH3; 2 mg/kg total) was delivered to groups of female BALB/c mice by the intranasal route 1 h before RT challenge. In the pilot study, the PB10/SylH3 cocktail showed a notable benefit over PB10 alone, as evidenced by reduced weight loss over the two-week period following RT challenge (Figure 1).

In separate studies, we have identified a large collection of single-domain antibodies (V_H_Hs) specific for RTA or RTB, including some with highly potent toxin-neutralizing activity in vitro [20]. While individual V_H_Hs have limited in vivo toxin-neutralizing activity [21], V_H_H cocktails proved highly effective at protecting Kupffer cells, they were not as effective as PB10/SylH3 in the intranasal challenge model (data not shown).

**Figure 2:**
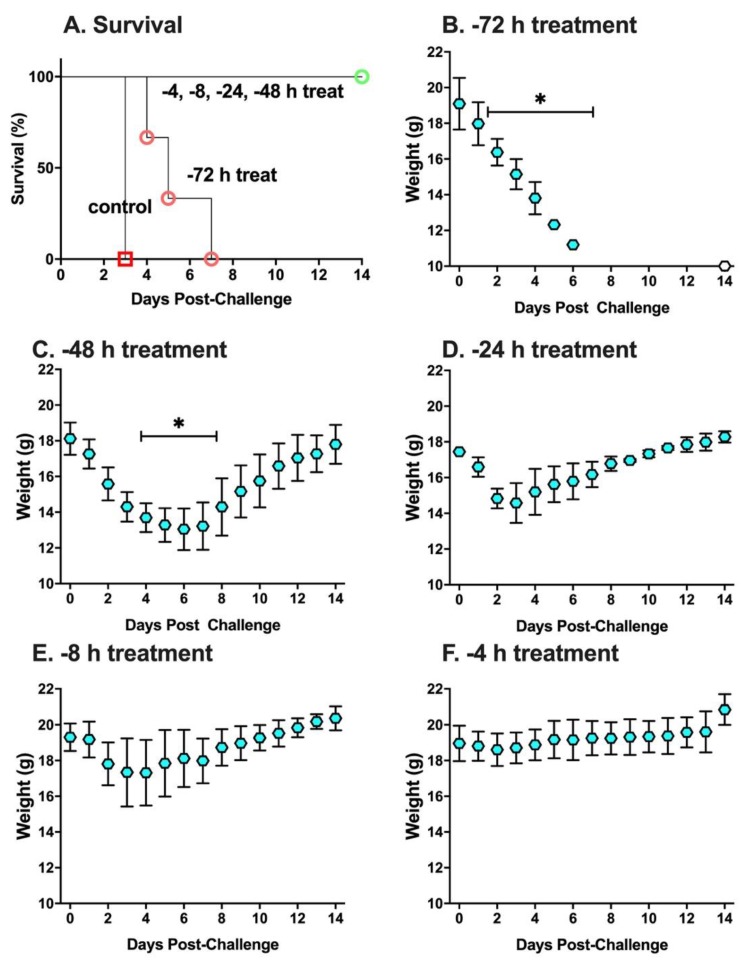
Prophylactic potential of the PB10/SylH3 cocktail in a mouse model of intranasal ricin challenge. Groups of BALB/c mice (*n* = 3 per group) were administered the PB10/SylH3 cocktail (2 mg/kg) by the intranasal route at the time points indicated (−72, −48, −24, −8 and −4 h) prior to 10 x LD_50_ RT challenge by the same route. The mice were then monitored for (**A**) survival and (**B**–**F**) weight loss for a two-week period. The RT group received RT without antibody, while the control group received vehicle only (saline). For the treatment groups, each mouse received a total of 40 µg of antibody (20 µg PB10 plus 20 µg SylH3 for the cocktail; 40 µg of PB10 alone). (Panel **A**) Kaplan-Meier survival plot. Only animals in the RT only (red square) and −72 h treatment groups (red circle) succumbed to ricin intoxication. All other animals survived RT challenge (overlapping green circle) although mice in the −48 h treatment group displayed hunching and solitary nesting (clinical score of 2), and mice in the −24 and −8 groups had ruffled fur (clinical score 1). (Panels **B**–**F**) Weight per day per group (average with SEM). Statistical analysis of weight loss (* indicates significant loss compared to pre-challenge values) was performed using Friedman tests with Dunn’s multiple comparison tests. During the course of the study, mice were weighed daily and visually inspected twice daily.

To better define the prophylactic window afforded by the PB10/SylH3 MAb cocktail, the MAb combination (2 mg/kg) was administered to groups of mice by the intranasal route at five different time points (−72, −48, −24, −8 and −4 h) prior to RT challenge. Mice were then monitored for 14 days for survival (mortality) and weight loss (morbidity). In terms of mortality, all groups of mice treated with the PB10/SylH3 MAb cocktail survived RT exposure, except for the −72 h-treatment group, which succumbed to intoxication within 3 days (Figure 2A). In terms of morbidity, the mice treated with PB10/SylH3 MAb at −72 h experienced significant weight loss at day 3 post-challenge, while weight loss on subsequent days was not compared statistically due to mortality. The −48 h group also showed significant weight loss on days 4–7, whereas the other three groups of animals (−24 h, −8 h, −4 h treatments) displayed no significant weight loss on any day (Figure 2B–F). Non-parametric Friedman tests with Dunn’s post hoc tests were used to compare weights at time points following challenge with starting weights. We considered *p*-values of ≤ 0.05 (two-tailed tests) to be statistically significant.

**Figure 3:**
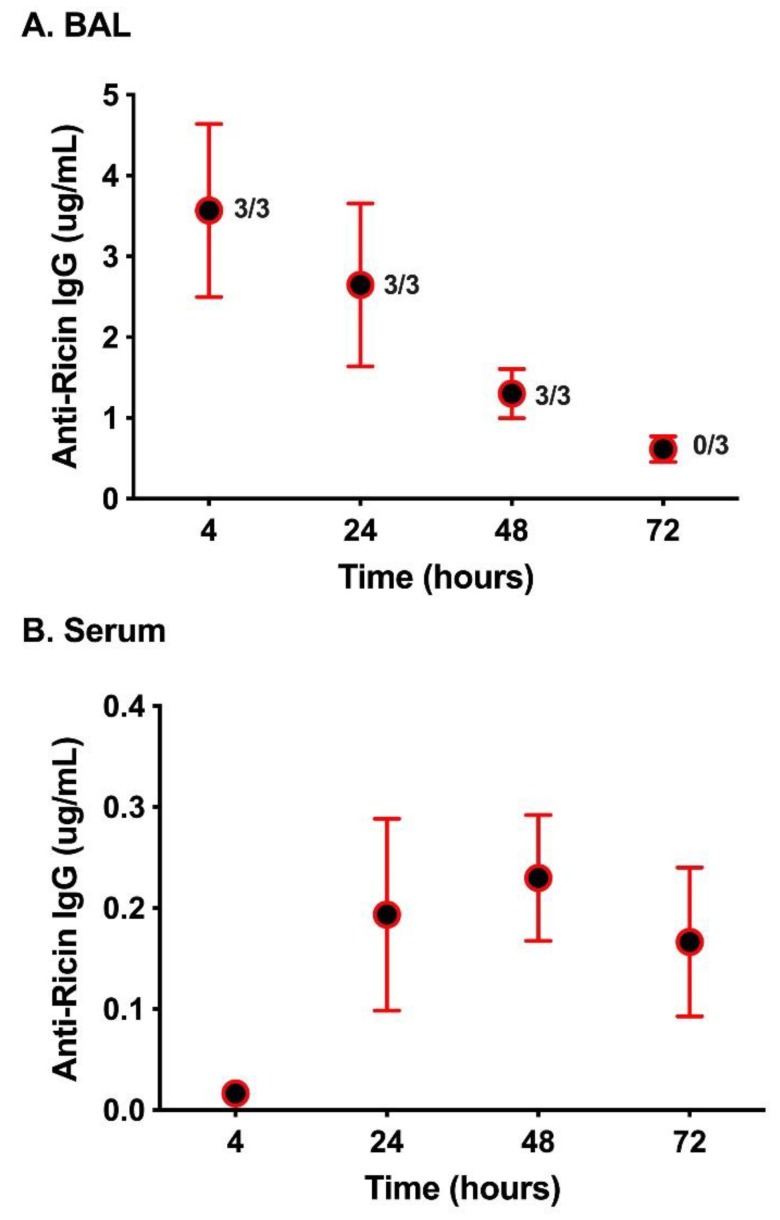
PB10/SylH3 levels in BAL fluid and serum following intranasal instillation in mice. Groups of mice (*n* = 3) were administered the PB10/SylH3 cocktail (2 mg/kg) by the intranasal route. (**A**) BAL fluids and (**B**) serum samples were collected from groups of animals at the indicated time points (4, 24, 48, 72 h) and then assessed for PB10/SylH3 levels by RT ELISA [10]. In panel A, the numbers adjacent to each symbol correspond to number of mice that survived per group (survivors/group) from Figure 1, where mice received same dose regimens of PB10/SylH3 cocktail as in this figure except that they were subsequently challenged with RT.

The efficacy of the cocktail coincided with the relative levels of PB10/SylH3 in the lung. Specifically, in a parallel study, BAL fluids (and sera) were collected from mice at fixed intervals (+4, +24, +48, +72 h) after MAb administration and evaluated by RT-specific ELISA. The results revealed an estimated antibody half-life in the BAL fluids of ~18 h (Figure 3A). In serum, low levels (0.1–0.3 µg/mL) of PB10/SylH3 MAb were detected at the +24 h timepoint and persisted until at least 72 h (Figure 3B). Taken together, the results suggest a local threshold concentration of >1 μg/mL of PB10/SylH3 is required to fully protect mice against the effects of pulmonary RT exposure.

The benefit of the PB10/SylH3 MAb cocktail over PB10 alone in the PrEP model incentivized us to produce a humanized variant of SylH3 that could be paired with huPB10 for eventual testing in NHPs [12]. The chimeric mouse Fv-human IgG1 Fc variant of SylH3 constructed several years ago was used as the starting material [22]. Candidate humanized SylH3 MAbs were first generated computationally using the Molecular Operating Environment software, which surveyed the Protein Data Bank (PDB) for the best fit human structures. Humanized variants were then further evaluated in silico for a variety of features that impact assembly and expression (e.g., methionine oxidation, asparagine deamidation, glycosylation, etc.). Based on these computational criteria, we generated expression vectors for nine candidate light chains and nine candidate heavy chains and expressed 81 unique antibody combinations in the RAMP system. The combinations demonstrating sufficient expression levels necessary for future scale-up were then tested in vitro. A total of eight Fv-humanized SylH3 candidates met the express level threshold and were evaluated for binding affinity by surface plasmon resonance (SPR; Figure S1) and in vitro toxin-neutralizing activity in Vero cell cytotoxicity assay (Table 1). Based on binding affinity, toxin-neutralizing activity, and pilot production yields, a single variant, huSylH3 07/03, was chosen as the lead molecule.

**Figure 4:**
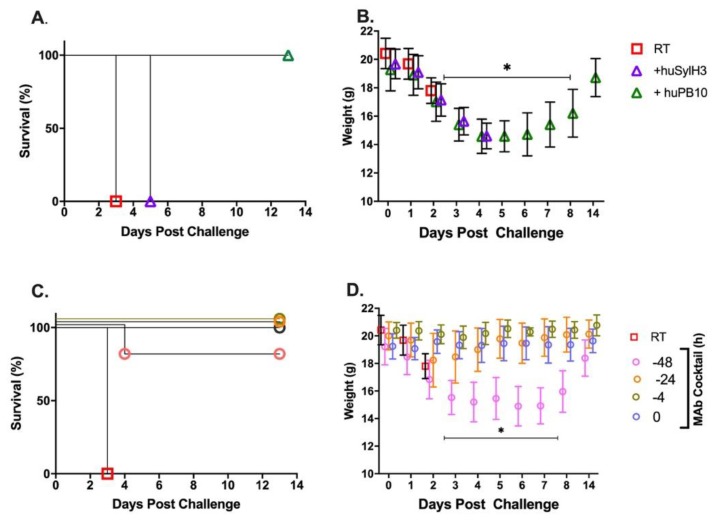
PrEP with the huPB10/huSylH3 cocktail protects mice against lethal-dose intranasal ricin challenge. (Panels **A**,**B**) Groups of BALB/c mice (*n* = 5 mice/group) were administered RT in vehicle RT plus huPB10 (green triangle) or RT plus huSylH3 at time 0. (Panels **C**,**D**) Groups of mice (*n* = 5 mice/group) received huPB10/huSylH3 cocktail at the indicated time points prior to RT challenge. MAbs and the MAb cocktails were given at a final dose of 2 mg/kg by the intranasal route. Mice were challenged with 10 x LD_50_ RT and then monitored for 14 days. (Panels **A**,**C**) Kaplan-Meier survival plots. (Panel **B**,**D**) Weight per day per group (average with SEM). Statistical analysis of weight loss was performed using Friedman tests with Dunn’s multiple comparison tests. In panel B, mice that received RT plus huPB10 displayed ruffled fur, hunching, ataxia, weakness, and/or reduced movement (clinical score, 2). Mice that received RT plus huSylH3 displayed severe weakness, tremors, head tilt, seizures (clinical score, 3) and were euthanized. In panel D, the group of animals that received the cocktail at −48 h (pink circles) experienced significant weight loss on days 4, 6, and 7 post-challenge (as indicated by asterisk and horizontal bar) and a single mouse succumbed to intoxication. The mouse that succumbed to challenge on day 4 was excluded from that point forward from the weight loss analysis. During the course of the study, mice were weighed daily and visually inspected twice daily.

To evaluate the huSylH3/huPB10 cocktail in vivo, the MAbs (2 mg/kg) were administered intranasally to mice before (−48, −24, −4 h) they were subjected to a 10 x LD_50_ RT challenge (Figure 4). As a control, groups of mice received huSylH3 (2 mg/kg), huPB10 (2 mg/kg) or the combination of huSylH3 and huPB10 (2 mg/kg) concurrently with RT (*t* = 0). The results revealed that the humanized MAb cocktail performed as well as the mouse MAb cocktail. The RT only group lost a significant amount of weight by day 2 post-challenge and expired by day 3. The groups of mice that received the huSylH3/huPB10 cocktail at −24 h, −4 h, or time 0 survived the RT challenge and did not experience any significant weight loss in the subsequent two weeks. Only the group of animals that received the cocktail at −48 h experienced significant weight loss (days 4, 6, and 7) post-challenge, with all but one of the mice recovered by day 14. By way of comparison, mice that received RT plus huSylH3 IgG1 (2 mg/kg) alone succumbed to RT intoxication by day 5, while the group of animals that received huPB10 alone survived the RT challenge, although they lost a significant amount of weight on days 3–7. These results demonstrate the capacity of the humanized huSylH3/huPB10 MAb cocktail to neutralize RT in a mouse model of pulmonary toxin exposure.

## 3. Discussion

RT remains a biothreat agent of concern to civilians and military personnel alike, due to its capacity to elicit debilitating and possibly fatal pulmonary inflammation [1]. Following inhalation, RT triggers hemorrhage, inflammatory exudate, and elevated levels of pro-inflammatory cytokines like IL-6, IL-1 and tumor necrosis factor alpha (TNF-α), which coincide with an influx of polymorphonuclear cells (PMN) and overall disease severity [3,5,8,11,12]. The underlying pathophysiology of RT is the consequence of ricin’s two subunits, RTA and RTB, working in concert to promote programed cell death in different cell types. RTB promotes toxin uptake into mammalian cells by at least two distinct pathways [23], while RTA inactivates ribosomes with near perfect efficiency [24]. Alveolar macrophages are particularly sensitive to RT and their numbers plummet within hours of toxin exposure [7,10,25]. Mucosal damage is exacerbated by TNF-α and its family members like TNF-related apoptosis-inducing ligand (TRAIL), which render lung epithelial cells hyper-sensitive to the effects of RT [9,26].

In a previous report, we demonstrated in a mouse model that intranasal delivery of a bipartite MAb cocktail, consisting of murine PB10 IgG, targeting RTA, and murine SylH3 IgG, targeting RTB, afforded near complete protection against a lethal-dose intranasal RT challenge [10]. Mice that received PB10/SylH3 cocktail concurrently with RT did not experience weight loss or have any significant lung inflammation, as measured by histopathology and pro-inflammatory cytokine production (IL-1, IL-6). The PB10/SylH3 mixture was also highly effective at shielding alveolar macrophages from ricin-induced killing, which we postulate is due to the fact that MAbs are present locally within the lung environment. As a post-exposure therapeutic, the murine PB10/SylH3 cocktail had activity that exceeded that of either of the MAbs alone.

In the current report, we have now demonstrated that the murine PB10/SylH3 cocktail is beneficial when delivered to mice locally (intranasally) before RT challenge. Based on these results, we produced a humanized version of SylH3 that proved to have in vivo toxin-neutralizing activity when paired with a humanized variant of PB10 that had already been evaluated in Rhesus macaques [12,13]. Ultimately, we are interested in the prospect of developing a self-administered (inhalable) immunoprophylactic capable of conferring immediate immunity to RT, which could be used in the field by military or civilian first responders. As noted in the introduction, Respaud and colleagues have already conducted proof-of-principle studies and successfully delivered an RT-specific neutralizing MAb to mice with the goal of preventing/treating pulmonary intoxication [19]. There are obvious technical challenges associated with this route of delivery, including distinct differences in IgG half-lives within the context of the lung, which have been noted even when the MAbs that have the same Fc domains [27,28,29,30]. The apparent differences in half lives were attributed to non-specific tissue binding or poor stability dictated in part by the Fv elements. Nonetheless, the fundamental biology and technology associated with aerosol delivery of MAbs could be applied in theory to combatting other toxins or infectious agents that cause severe acute respiratory disease, including the new coronavirus (SARS CoV-2).

The mechanism(s) by which PB10 and SylH3 work to neutralize RT is an area of active investigation. PB10 is directed against an immunodominant epitope on RT’s enzymatic subunit (RTA), while SylH3 is against an epitope localized to RTB’s domain 1 (Vance, D.; Poon, A. and Mantis, N. manuscript in preparation). SylH3′s in vitro profile is similar to many other anti-RTB MAbs that have been described in the literature, although it stands out because it is the most effective MAb in our collection at blocking RT-receptor interactions in vitro [31]. We hypothesize that the PB10/SylH3 cocktail has dual activities: it blocks RT attachment to host cells and it interferes with retrograde transport of ricin from the plasma membrane to the TGN [31,32]. We hypothesize that the presence of huPB10 and huSyH3 in the lung at the time of ricin challenge results in antibody-mediated entrapment of the toxin within the alveolar space. This hypothesis is based on a recent study in mice with the anti-Ebola antibody cocktail ZMapp, which indicated that the antibodies did indeed limit accessibility and mobility of the virus in the airways [33].

There are a large number of RT-specific MAbs from mice and NHPs described in the literature that have been shown to be able to passively protect mice against RT challenge [34,35,36,37,38,39,40,41,42]. It is unclear whether the PB10/SylH3 cocktail is any more potent that other possible combinations of MAbs described, since systematic comparisons have not been conducted, although certainly the two MAbs are the best within our collection [10]. Indeed, it would be of interest to the field to perform rigorous side-by-side passive protection studies in an effort to identify the MAb or combination of MAbs that is ultimately most effective at neutralizing RT within the lung.

An interesting aspect of the PB10/SylH3 cocktail which is being explored in a separate study is its capacity to stimulate active immunity to RT. Specifically, we have observed that RT-PB10/SylH3 immune complexes given to mice by the intranasal route elicit long-lasting toxin-neutralizing antibody responses that persists for months (Tolman, L.; Yates, J.; Rong, Y. and Mantis, N. manuscript in preparation). The phenomenon is reminiscent of a so-called “vaccinal effect” in which immune complexes prime both cellular and humoral immunity following a Fc-receptor-dependent antigen sampling by dendritic cells [43]. Thus, the PB10/SylH3 cocktail may have a dual benefit, in conferring passive immunity as well as eliciting active long-lasting anti-toxin immunity.

## 4. Materials and Methods

### 4.1. Chemicals and Biological Reagents

RT (*Ricinus communis* agglutinin II) was purchased from Vector Laboratories (Burlingame, CA, USA). RT was dialyzed against phosphate buffered saline (PBS) at 4 °C in 10,000 MW cutoff Slide-A-Lyzer dialysis cassettes (Pierce, Rockford, IL, USA) prior to use. Unless noted otherwise, all other chemicals were obtained from Sigma-Aldrich (St. Louis, MO, USA).

### 4.2. Monoclonal Antibodies (MAbs)

Murine MAbs against RTA (PB10) and RTB (SylH3) were purified using ion-exchange and protein G chromatography, as described [14,31]. Humanized PB10 (huPB10) and the SylH3 (huSylH3) were each expressed in a *Nicotiana benthamiana*-based rapid-antibody manufacturing platform (RAMP) [16,17].

### 4.3. Ethics Statement for Studies Involving Mice

Mouse studies were conducted under strict compliance with the Wadsworth Center’s Institutional Animal Care and Use Committee (IACUC) under approval code ^#^18-384 on 20 December 2018 for a period of 3 years. The Wadsworth Center complies with the Public Health Service Policy on Humane Care and Use of Laboratory Animals (Assurance ^#^A3183-01). The Wadsworth Center is fully accredited by the Association for Assessment and Accreditation of Laboratory Animal Care (AAALAC). Obtaining this voluntary accreditation status reflects that Wadsworth Center’s Animal Care and Use Program meets all standards required by law, and goes beyond the standards as it strives to achieve excellence in animal care and use. Mice were euthanized by carbon dioxide asphyxiation followed by cervical dislocation, as recommended by the Office of Laboratory Animal Welfare (OLAW), National Institutes of Health.

### 4.4. Experimental Design of Animal Studies

Female BALB/c mice (ages 8–10 weeks) were purchased from Taconic (Rensselaer, NY). For acute exposures, PB10 (40 μg), SylH3 (40 μg), or the combination (20 µg PB10 + 20 µg SylH3) were administered by the intranasal (i.n.) route to mice in a total volume of 40 µL at time points before 10 x LD_50_ RT challenge. The LD 50 dose was determined empirically and corresponds to 10 μg/kg. During the course of a study, mice were weighed once daily and visually inspected twice daily for signs of morbidity. Visual inspections were done using a grading sheet approved by the IACUC. We recorded and graded signs of hunching, mild to moderate weakness, ataxia, distended abdomen, diarrhea, solitary nesting, ruffled fur severe weakness, tremors, circling, head tilt, seizures, and swollen eyes. Specifically, we employed a clinical scoring matrix to assess severity of morbidity following toxin challenge (Table 2). The scoring sheet was used along with weight loss as a means to provide quantitative criteria for determining when to euthanize an animal in the experimental protocol.

Mice were euthanized when their clinical score or weight loss exceeded a predetermined threshold of 3. The mice were euthanized by carbon dioxide asphyxiation, followed by cervical dislocation. The lungs were then lavaged with 1 mL of ice-cold PBS. Bronchoalveolar lavage (BAL) fluids plus cells were centrifuged at 3000 rpm at 4 °C for 10 min. Supernatants were collected and stored at −20 °C until analysis.

### 4.5. Vero Cell Cytotoxicity Assays

Vero cell cytotoxicity assays were performed as previously described [44,45]. Vero cells were detached from culture dishes with trypsin, then adjusted to ~5 × 10^4^ cells per ml, and seeded (100 µL/well) into white 96-well plates (Corning Life Sciences, Corning, NY, USA) and allowed to adhere overnight. The cells were then treated with ricin (0.01 µg/mL; 154 pM), ricin:MAb mixtures, or medium alone (negative control) for 2 h at 37 °C. The cells were washed and then incubated for 48 h, at which time cell viability was assessed using CellTiter-GLO (Promega, Madison, WI, USA). All treatments were performed in triplicate, and 100% viability was defined as the average value obtained from wells in which cells were treated with medium only.

### 4.6. ELISA Methodology

Nunc Maxisorb F96 microtiter plates (ThermoFisher Scientific, Pittsburgh, PA, USA) were coated overnight with ricin (0.1 µg/well; 15 nM), RTA (0.1 µg/well; 8 nM), or RTB (0.1 µg/well; 29 nM), before being treated with mouse sera, BAL fluids or MAbs. Antibodies were diluted in PBS, as necessary, horseradish peroxidase (HRP)-labeled goat anti-mouse IgG-specific polyclonal antibodies (SouthernBiotech, Birmingham, AL, USA) were used as the secondary reagent. The ELISA plates were developed using 3,3′,5,5′-tetramethylbenzidine (TMB; Kirkegaard & Perry Labs, Gaithersburg, MD, USA) and analyzed with a SpectroMax 250 spectrophotometer equipped with Softmax Pro 5.4 software (Molecular Devices, Sunnyvale, CA, USA).

### 4.7. Surface Plasmon Resonance (SPR)

Humanized SylH3 MAb association and dissociation rates in Table 1 were determined by surface plasmon resonance (SPR) using a Biacore T200 (GE Healthcare, Chicago, IL, USA). Sensor Chip Protein G was used for capturing antibody ligands. Humanized SylH3 MAbs, diluted in running buffer, were captured on the chip surface at a maximum of 100 RU at a flow rate of 10 μL/min). Serial dilutions of ricin, diluted in running buffer, were injected at a rate of 50 μL/min. After each injection, the chip surface was regenerated with 10 mM glycine-HCl pH 2.0 at 30 μL/min for 40–45 s. Sensorgrams were normalized by subtracting baseline RU values from a reference flow cell (absent capture MAb) and analyzed by fitting the data to the 1:1 Langmuir binding model using the Biacore T200 Evaluation Software 3.1 (GE Healthcare).

### 4.8. Humanization of SylH3

Humanized derivatives of SylH3 MAb were generated computationally using Molecular Operating Environment software (Chemical Computing Group, Montreal, PQ, Canada; https://www.chemcomp.com/Products.htm), which surveys the Protein Data Bank (PDB) (https://www.rcsb.org/) for the best fit human structures [46,47]. Humanized variants were then further evaluated in silico for a variety of features that impact assembly and expression, including methionine oxidation [48], asparagine degradation and/or deamidation [49,50,51], glycosylation, and aggregation prone domains. Based on these computational criteria, expression vectors for nine candidate light chains and nine candidate heavy chains were constructed, after which 81 unique antibody combinations were tested in the RAMP system. Those combinations demonstrating adequate expression for future scale-up were then further narrowed down in a ricin binding assay using an Octet and Bio-Layer Interferometry (ForteBio, Fremont, CA, USA).

### 4.9. Statistical Analyses

Non-parametric Friedman tests with Dunn’s post hoc tests were used to compare weights at time points following challenge with starting weights. We considered *p*-values of <0.05 (two-tailed tests) to be statistically significant. Statistical analyses were performed using GraphPad Prism 8 (GraphPad Software, San Diego, CA, USA).

## Figures and Tables

**Table 1:** Characteristics of humanized SylH3 variants.

Hu Variant	*k_a_* (1/Ms)	*k_d_* (1/s)	*K_D_* (M) *^a^*	EC_50_*^b^*	IC_50_*^c^*
1008	1.25 × 10^6^	6.78 × 10^−5^	5.42 × 10^−11^	0.15	2
0403	1.33 × 10^6^	7.3 × 10^−5^	5.45 × 10^−11^	0.078	1
**0703**	**1.35 × 10^6^**	**7.79 × 10^−5^**	**5.74 × 10^−11^**	**0.019**	**1**
0107	1.59 × 10^6^	3.81 × 10^−5^	2.39 × 10^−11^	0.078	2
0206	1.41 × 10^6^	1.37 × 10^−4^	9.96 × 10^−11^	0.039	1
0908	1.34 × 10^6^	7.29 × 10^−5^	5.43 × 10^−11^	0.039	1
0801	1.46 × 10^6^	7.45 × 10^−5^	5.07 × 10^−11^	0.039	1
0209	1.40 × 10^6^	1.35 × 10^−4^	9.62 × 10^−11^	0.019	1

^a^ Apparent affinity or avidity; ^b^ MAb (μg/mL) required to achieve half-maximal binding to RT by indirect ELISA; ^c^ MAb (μg/mL) required to neutralize 50% RT (10 ng/mL) in Vero cell cytotoxicity assay, as described in the materials and methods section. Bold, indicates MAb variant chosen for in vivo studies.

**Table 2:** Scoring system for mice inoculated with RT.

Score	Clinical Signs
0	Normal
1	Ruffled Fur
2	Hunching, ataxia, distended abdomen, diarrhea, solitary nesting,
3	Severe weakness, tremors, circling, head tilt, seizures, eyes closed
4	Paralyzed limb(s)
5	Morbid, non-responsive
6	Dead

## Supplementary Materials

The following are available online at https://www.mdpi.com/2072-6651/12/4/215/s1, Figure S1: Representative sensorgrams of humanized SylH3 variants.
