# Supplementary material for: A Humanized Monoclonal Antibody Cocktail to Prevent Pulmonary Ricin Intoxication
Source: Toxins (Basel). 2020 Mar 29;12(4):215. doi: 10.3390/toxins12040215 (PMC7232472; doi:10.3390/toxins12040215)
Supplement: Supplementary file 1 [file toxins-12-00215-s001.pdf]

Supplementary Materials: A Humanized Monoclonal Antibody Cocktail to Prevent Pulmonary Ricin Intoxication

Yinghui Rong, Michael Pauly, Adrian Guthals, Henry Pham, Dylan Ehrbar, Larry Zeitlin and Nicholas J. Mantis *

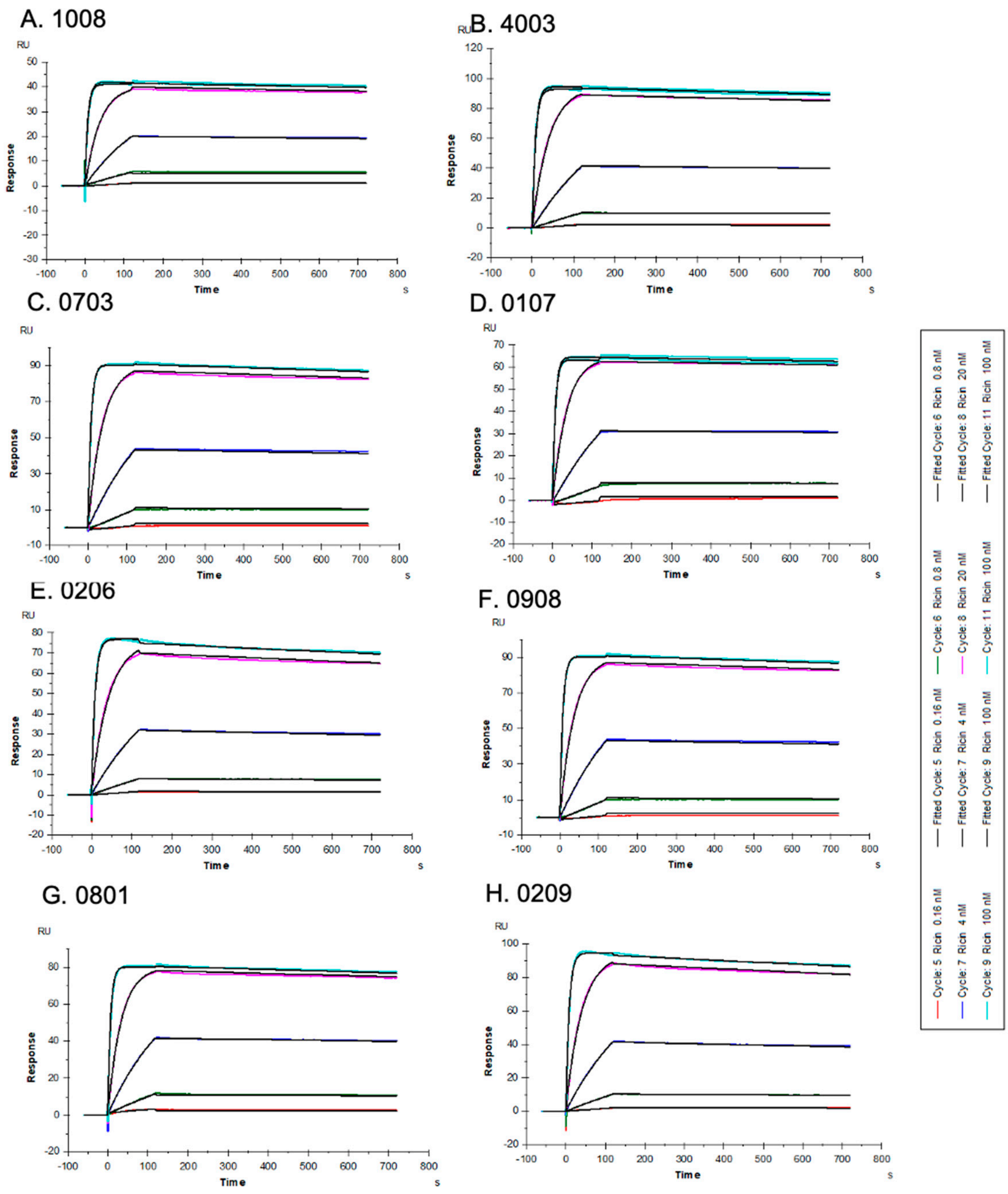

Figure S1. Representative sensorgrams of humanized SylH3 variants. Humanized SylH3 MAb candidates (panels **A**–**H**) diluted in running buffer were captured on Sensor Chip protein G, as described in Materials and Methods. Ricin was injected at the indicated concentrations (dark turquoiose, 100nM; fuchsia, 20 nM; blue, 4 nM; dark green 0.80 nM; red, 0.16 nM) at a flow rate of 50 μL/min in running buffer. The chip surface was regenerated at pH 2.0 for 40–45 s following each ricin injection. Kinetic constants for the antibody/ricin interactions were obtained with Biacore T200 Evaluation Software 3.1 using the 1:1 Langmuir fit model.
